# Influence of Refractive Index on Antireflectance Efficiency of Thin Films

**DOI:** 10.3390/ma12091483

**Published:** 2019-05-07

**Authors:** Sadaf Bashir Khan, Syed Irfan, Zheng Zhuanghao, Shern Long Lee

**Affiliations:** 1 Institute for Advanced Study, Shenzhen University, Shenzhen, Guangdong 518060, China; sadafbashirkhan@szu.edu.cn; 2 Key Laboratory of Optoelectronic Devices and Systems of Ministry of Education and Guangdong Province, College of Optoelectronic Engineering, Shenzhen University, Shenzhen 518060, Guangdong, China; syedirfan@szu.edu.cn (S.I.); zhengzh@szu.edu.cn (Z.Z.); 3 Shenzhen Key Laboratory of Advanced Thin Films and Applications, College of Physics and Energy, Shenzhen University, Shenzhen 518060, China

**Keywords:** refractive index, antireflective, thin film, wavelength, simulation, substrate

## Abstract

In today’s world, scientific development is tremendously strengthened by imitating natural processes. This development remarkably validates progressive and efficient operation of multifunctional thin films in variable ecological circumstances. We use TFCalc thinfilm software, a reliable and trustworthy simulation tool, to design antireflective (AR) coatings for solar cells that can operate in varying environmental conditions and can be functional according to user-defined conditions. Silicon nearly reflects 36% light in the 550 nm wavelength region, causing a significant loss in solar cell efficiency. We used silicon as the substrate on which we designed and fabricated a trilayer inorganic oxide AR thin films, and this reduced it reflectance to <4% in the 300~800 nm wavelength range. Because of their distinguishing physical physiognomies, we used a combination of different inorganic oxides, comprising high-, low-, and medium-refractive indices, to model AR coatings in the desired wavelength range. Experimental implementation of the designed AR thin films in the present study unlocks new techniques for production of competent, wideband-tunable AR coatings that are applicable in high-performance photovoltaic applications.

## 1. Introduction

The threat of approaching energy deficiency is making it progressively more imperative to advance new, alternative substitutes for energy harvesting equipment. Present-day solar cell fabrication is dominated by crystalline silicon modules because of their established production procedures and comparatively high efficiency [1,2]. The working efficiency and productivity of silicon (Si) solar cells have been restrained because of the high refractive index of Si (η = 3.4 at 550 nm) [3]. More than 40% of the incident light is reflected and generates reflectance losses, which significantly lessens the efficiency of the photovoltaic device [4,5]. Different approaches were used to overcome this optical loss by trapping light to enhance Si solar cell efficiency. Numerous procedures have been industrialized, such as texturing Si structures using dry or wet etching processes or fabricating antireflective composite thin films [6,7,8]. However, the majority of these common texturing methods are either too expensive or possess too low replicability to advance the progress of solar cells in an economically reasonable way [5,8,9,10].

Recently, in high-tech applications use of metal nanostructures has remarkably grown because of their distinguishing chemical and physical properties. The surface plasmonic resonance (SPR) of metallic nanostructures can be easily varied by changing geometrical dimensions, morphology, and surface chemistry to enhance the solar cell working efficiency [11,12]. Incorporation of metallic particles in Si and GaAs solar cells increases output productivity [13,14,15]. The major drawback of embedded metallic nanostructures is that they form electron-hole (e–h) pair recombination centers. Lim enhanced the photocurrent efficiency of an Si photodiode up to 3% by embedding Au particles [16].

We used the thin film Calc software (TFCalc) method to analyze and stimulate the optical behavior of different inorganic oxide antireflective (AR) coatings on a silicon substrate in the visible regime to obtain low-reflection conditions. The amorphous silicon solar cells undergo deterioration, intrinsic instability, and lowered proficiency over time. However, if AR coatings were fabricated on silicon solar cell modules, then one could enhance its efficiency and long-term durability for a wide range of applications of photovoltaic devices. AR coatings not only help in trapping incident omnidirectional light but also play an important role in surface passivation. Relatively favorable and practically applicable AR coating simulations have been obtained using inorganic oxides for monolayer, bilayer, or multilayer AR coating designs. We simulated HfO_2_ as an AR film on silicon because of its high hardness, mechanical strength, and transparency in the infrared to ultraviolet region [17,18]. In addition, HfO_2_-encapsulated Al_2_O_3_ also showed good surface passivation, which was beneficial for solar cell devices [19]. Earlier, Kessels et al. experimentally proved that Al_2_O_3_ thin films (7–30 nm) manufactured via plasma-assisted atomic layer deposition exhibited excellent surface passivation of crystalline silicon [20]. Similarly, we also selected SiO_2_ and TiO_2_ to simulate AR coatings on silicon because of their semiconductive natures, hydrophilic characteristics, self-cleaning abilities, and thermal resistivities.

The purpose of the present work was to first design and fabricate robust AR coatings (comprising of single material system/composite AR coatings). Secondly, we investigated the influence of the refractive index and its impact on the AR efficiency of thin films under similar fabrication coatings using a physical vapor deposition technique. We studied the optical behavior of monolayer, bilayer, and trilayer AR coatings comprising high- and low-index material. We used SiO_2_, HfO_2_, and TiO_2_ to simulate AR coatings because of their potential applicability and distinctive physical characteristics. The current study pioneers a new direction for engineering efficient AR coatings used in high-performance and advanced optoelectronic and photovoltaic devices.

## 2. Materials and Methods

TFCalc (version 3.5, Software Spectra, Inc., Portland, OR, USA) is a computer software program that supported evaluating and scheming the monolayer, bilayer, or multilayer AR films, which encompassed single-component or amalgamated thin films [21]. This software played an influential role in optimizing the thickness of the individual layer in the coating stack to systematically modify the optical properties of the thin film. Diverse constraints could be figured out, calculated, adjusted, and designed including transmittance, reflectance, phase shift, or absorbance in the preferred wavelength region. The AR coating strategy includes a single stratum or multilayers; layers consolidate in group or assemblies may optimize according to ones need. TFCalc contributed in selecting the appropriate material *η*, the width of the distinct stratum, and the organization of individual layers to examine and simulate the AR performance of multilayer stacks in a coating scheme. 

Thick and thin strata were used for simulating and modeling AR films. The thick layer in the software was the one in which the phase statistics of reflected light from the forward-facing and the bottommost surface were lost because of the specified coating width. However, in thin films, light intrusion and phase statistics in the middle of the higher and lower surfaces were included. The contributing input factors for modeling the AR coating involved the simulated wideness of the discrete stratum, substrate η, wavelength, light incidence angle, and adjacent media *η*. The outcomes included AR coating transmittance or reflectance arcs. One can practically fabricate AR coatings based on the simulation parameters. Diverse inorganic, dielectric metals or metal oxides can be castoff to develop AR films. We used SiO_2_ (η~1.46), [22] HfO_2_ (η~1.9) [23], and TiO_2_ (η~2.4) [24] for simulating AR coatings comprising single layer, bilayer, and multilayer coatings. We discussed in detail the simulation parameters and the importance of the AR coating. The suggested principles could be applied to progress AR performance of optical assemblies, potentially leading to more efficient solar cells, windows, or integrated optical structures.

Experimental fabrications of AR thin films were carried out in a high-vacuum e-beam evaporation system with a base pressure of 3 × 10^−4^ Pa. A Quartz-crystal microbalance located close to the substrate holder was used to control the thickness of the layer. The thin films were deposited in a single phase to avoid any foreign contamination. In the case of bilayer/trilayer AR fabrications, after attaining an applicable thickness of the individual layer, the target holder was changed according to the requirements. The deposition rate was maintained at 5 A°/s during the preparation of thin films. Supporting substrates (silicon) were fixed on the substrate holder positioned at normal away from the target material. In our design strategy, the AR coatings were designed and formulated on the front-exposed surface.

Field emission scanning electron microscopy (FESEM) (JEOL-7001F, JEOL, Peabody, MA, USA) was used to analyze the cross-sectional morphology of composite thin films. A WVASE32 spectroscopic ellipsometer (JA Woollam Co., Inc, Lincoln, NE, USA) was used to determine the refractive indices of monolayer thin films in the wavelength range of 300–1000 nm. Reflectance measurements were performed via an angle-resolved microscope (ARM) series (HL2000 Pro, Ideaoptics instrument Co., Ltd, Shanghai, China) at normal light incidence.

## 3. Results

**Figure 1:**
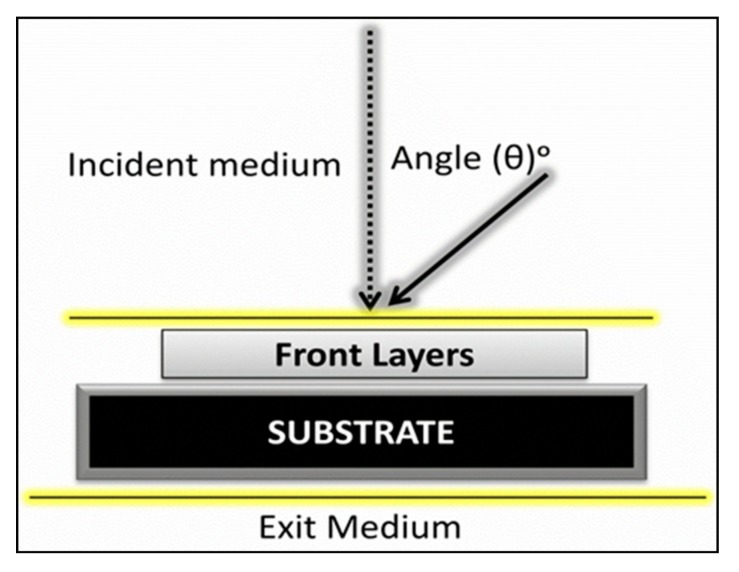
Schematic environmental conditions for the modeling of antireflective (AR) coatings on silicon.

In the present work, we used silicon as a supporting substrate, operating in the wavelength region (300~800 nm) in air media, and used white light as an illuminant because white light is colorless and analogous to ordinary daylight. It comprised all the wavelengths of the visible spectrum at an equal intensity. Figure 1 represents the schematic diagram of our simulated coating model in which the back reflectance from the substrate or the bottom AR layer was not considered. The incident media was air, while the exit media was the antireflective coating and the supporting substrate itself. We did not assume any reflectance or transmittance from the bottom surface of the supporting substrate. The reference wavelength was designated as 550 nm in all simulations.

We used three different dielectric inorganic oxides, SiO_2_, HfO_2_, and TiO_2_, to simulate AR coatings on Si. We individually modeled single layers, bilayers, and trilayer AR thin films by optimizing the thickness of each stratum in the coating. We used low-refractive and high-refractive inorganic oxides to show that, by appropriately selecting and optimizing the thickness, one could easily fabricate broadband AR coatings according to the desired wavelength region or optoelectronic device requirements. 

**Figure 2:**
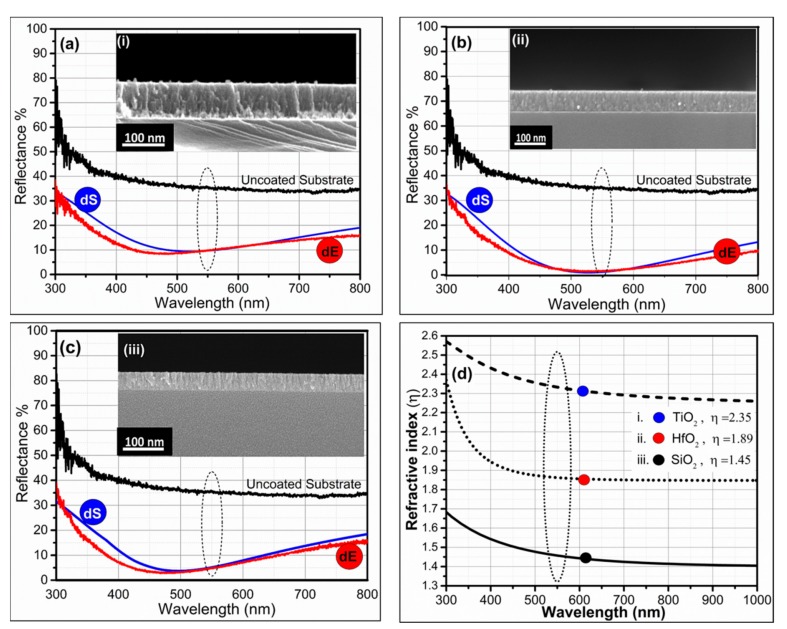
Experimental fabrication and simulation of single-layer AR coatings on silicon substrate (**a**) SiO_2_, (**b**) HfO_2_, and (**c**) TiO_2_. (The red curve represents the experimental reflectance (dE) curve, the blue curve represents the simulated reflectance (dS) of the model thin film, and the black curve represents the measured reflectance curve of the uncoated silicon substrate). (**d**) Refractive index of monolayer SiO_2_, HfO_2_, and TiO_2_ coatings as a function of wavelength.

Figure 2 represents the simulation and the experimentally fabricated reflectance results of the single layer AR coating. It was observed that a low-refractive index (η) inorganic oxide coating (i.e., SiO_2_) showed poor AR efficiency compared to high-η dielectric material (i.e., TiO_2_ and HfO_2_). Silicon had an η of nearly 3.4, and SiO_2_ had an η of 1.46 (Figure 2d) in the 550 nm wavelength region. Our experimental results showed that a dense SiO_2_ layer, with a thickness of 85~90 nm, could reduce the reflectance of silicon from 33.97% to nearly 10%~13% in the 500~700 nm wavelength region (Figure 2a). There was a sharp decrement of η from air towards the substrate because SiO_2_ showed poor AR efficiency in comparison with HfO_2_ or TiO_2_ AR coatings. The experimentally measured AR efficiency of thin films was analogous to simulated reflectance curves because simulated parameters were used to fabricate AR thin films. In Figure 2, dS demonstrated the simulated reflectance curve, and dE displayed the measured reflectance efficiency of fabricated thin films.

However, when HfO_2_ dense film was modeled on silicon, it showed <4% reflectance in the visible region of 500~600 nm. At 550 nm wavelength, HfO_2_ film, with a thickness of 50~60 nm, showed reflectance of nearly 1%–2%. It was highly desirable for solar cell devices. The HfO_2_ AR thin compact film showed good results because there was a gradual decrement of η from top to bottom, i.e., air~1 < HfO_2_~1.9 < Si~3.4 (Figure 2d). The experimental fabrication of HfO_2_ thin films, with a thickness of 55 nm according to simulation parameters, practically reduced the reflectance of silicon to <3% in the region of 550 nm, as shown in Figure 2b.

Similarly, simulation of TiO_2_ thin film on silicon showed reflectance up to 3%~7% in the 500~700 nm wavelength range. The fabrication of TiO_2_ thin film with a thickness of 45 nm on silicon reduced the reflectance of silicon from 36% to less than 5% at 550 nm wavelength. The experimental and simulated reflectance curves were nearly alike, as shown in Figure 2c. Overall, the dielectric monolayers showed good AR efficiency compared to the uncoated silicon. 

The experimental results showed that among all, HfO_2_ monolayer films were more appropriate (represented by an intermediate η~1.9 Figure 2d) on silicon compared to SiO_2_ or TiO_2_. SiO_2_ possessed a very low η compared to silicon (air~1 < SiO_2_~1.46 < Si~3.4), while TiO_2_ showed a very high and sudden sharp increase in η compared to the surrounding media (air~1 < TiO_2_~2.4 < Si~3.4). The experimentally measured and simulated reflectance curves are presented in Figure 2, and the percent reflectance, corresponding to wavelengths with appropriate thicknesses, is mentioned in Table 1. Previously, Kim and colleagues experimentally proved that in solar cell devices, the presence of HfO_2_ thin films increased the diffusion coefficient (D_e_) and lifetime (τ_e_) of photoelectrons. It helped in hindering back electron transfer, which upsurged the short-circuit current (J_sc_) and open-circuit voltage (V_oc_). Thus, solar cell photoconversion efficiency (η) was significantly enhanced, from 5.67% to 9.59% (an improvement of 69.02%), when the HfO_2_ layer was coated over TiO_2_ films [25]. Similarly, in graphene/silicon (Gr/Si) solar cells, the power conversion efficiency was remarkably enhanced via incorporating a thin film of HfO_2_ (from 3.9% to 9.1%). The acquired output with HfO_2_ interfacial layers developed by atomic layer deposition was considered among the highest reported for Gr/Si solar cells [26]. These results indicated that HfO_2_ was a promising candidate for future solar cells devices, as it acted as an antireflective thin film, directing maximum transmittance as well as increasing solar cell power conversion efficiency. 

**Figure 3:**
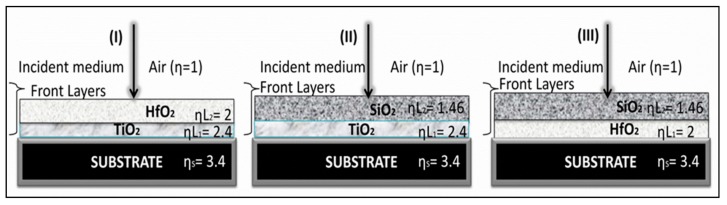
Schematic representations of a composite coating; a high-index and a low-index layer in a bilayer coating stack.

We also simulated and fabricated a bilayer composite AR coating to reduce the reflectance of uncoated silicon. Silicon reflected nearly 36%–33% in the 300~800 nm wavelength region, as shown in Figure 2 (black line). We simulated three composite coatings, by grouping together hh-index and low-index layers in a coating stack, as shown in schematic diagram Figure 3.

**Figure 4:**
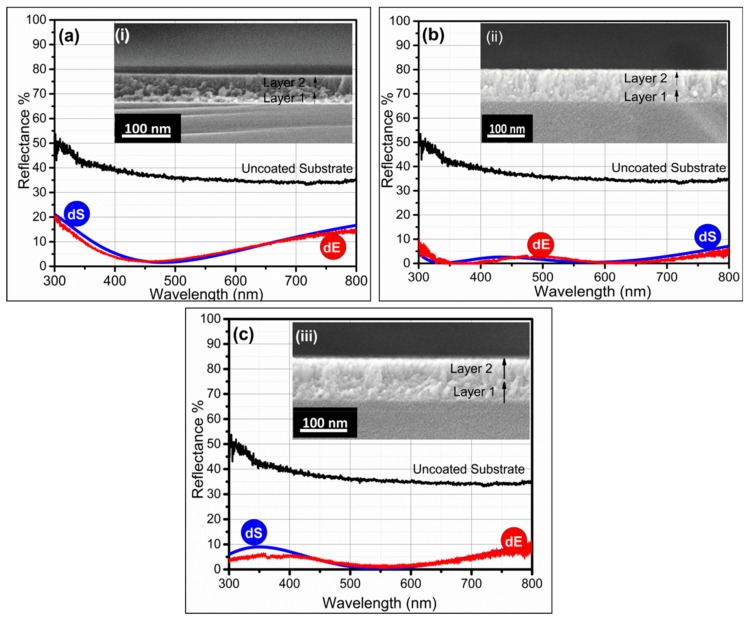
AR efficiency of bilayer composite AR coatings on silicon (**a**) TiO_2_-HfO_2_, (**b**) TiO_2_-SiO_2_, And (**c**) HfO_2_-SiO_2._

In all our simulations and experimental fabrications of bilayer coatings, ηL_1_ denotes layer 1 (adjacent to the substrate), and ηL_2_ denotes layer 2 in between air media and layer 1. Firstly, we grouped TiO_2_ and HfO_2_ into a coating stack and performed simulations to optimize the thickness of each stratum in a composite AR coating. Experimental results showed that by stacking the two high-dielectric inorganic oxides together, one could reduce reflectance 2%~4% in the wavelength range of 400~600 nm, as shown by the red curve in Figure 4a. The inset represents the SEM image of the bilayer composite TiO_2_-HfO_2_ thin film. The simulated (dS) and experimentally measured reflectance (dE) curves almost showed identical reflectances. Overall, the TiO_2_-HfO_2_ composite coating showed reduced reflectance compared to uncoated silicon (Figure 4a).

In the case of the TiO_2_-HfO_2_ AR coating, the declination of η from air towards substrate was η_S_ < ηL_1_ < ηL_2_ < η_AIR_, i.e., (3.4 < 2.4 < 1.9 < 1). The assembling of high- and low-η index material in a composite coating stack (TiO_2_-SiO_2_) showed efficient AR characteristics with reflectance <1% in the visible regions of 500~600 nm and 350 nm (Figure 4b). Assemblage of TiO_2_-SiO_2_ in a coating stack showed the best antireflective property, with a W-shaped curve, represented in Figure 4b. It clearly showed the measured reflectance was <4%–5% in the visible range. In the TiO_2_-SiO_2_ AR coating, there was a continuous steady and gradual decrement of η from the air towards the substrate, η_S_ < ηL_1_ < ηL_2_ < η_AIR_, i.e., (3.4 < 2.4 < 1.46 < 1), which was why it showed an enhanced AR performance. Similarly, stacking of HfO_2_-SiO_2_ coatings also showed good AR performance, as shown in Figure 4c. The HfO_2_-SiO_2_ composite coating showed a consistent declination in η such that η_S_ < ηL_1_ < ηL_2_ < η_AIR_, i.e., (3.4 < 1.9 < 1.46 < 1). Experimental results depicted that the coating showed <1% reflectance in the visible region of 500~600 nm (Figure 4c). Experimental results proved that HfO_2_-SiO_2_ and TiO_2_-SiO_2_, assembled in a composite coating formulation, were more applicable compared to the TiO_2_-HfO_2_ composite coating, as both (TiO_2_ and HfO_2_) were high-index materials. Effective and appropriate thicknesses of each layer in a bilayer coating stack in assembling composite coating and the percent reflectance in the visible region are mentioned in Table 2.

**Figure 5:**
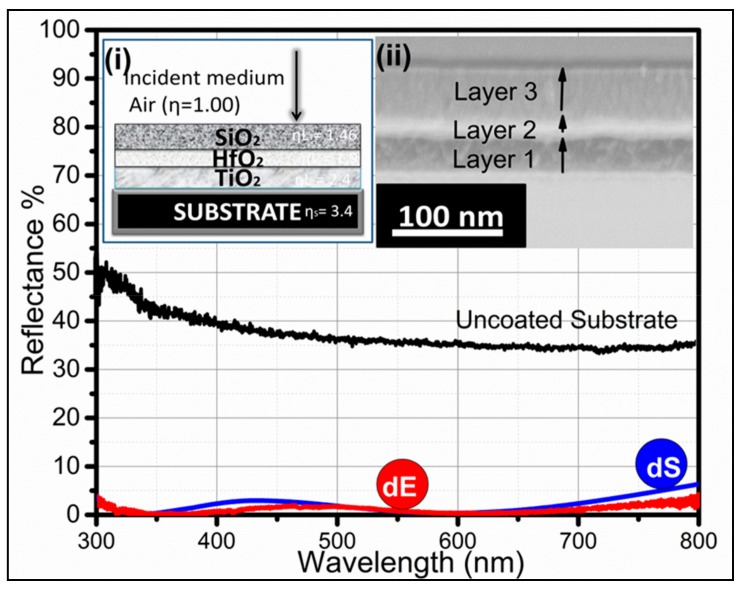
Simulated and measured AR efficiencies of trilayer AR composite coatings. The inset demonstrates (**i**) schematic representation of the trilayer AR coating and (**ii**) cross-sectional SEM image of the fabricated AR coating.

We also simulated and fabricated a trilayer composite AR coating using high-index (TiO_2_) low-index (SiO_2_) coatings with an inorganic HfO_2_ having η intermediate between the two. This composite AR coating showed good AR performance, with a reflectance 0.33%–3% in the wavelength range of 300~700 nm, compared to uncoated Si, as shown in Figure 5. Schematic representation of the trilayer AR coating and the cross-sectional SEM image of the fabricated AR coating are shown in the inset of Figure 5i,ii. The trilayer coating showed a continuing declination of η, which was η_S_ < ηL_1_ < ηL_2_ < ηL_3_ < η_AIR_, i.e., (3.4 < 2.4 < 1.9 < 1.46 < 1). There was steady change and a constant decrease in η from air towards the bottom substrate, as this coating stack showed good AR performance. The simulation and experimental parameters of the trilayer composite AR coating are displayed in Table 3 and Figure 5. Firstly, TiO_2_ was deposited, on top of which HfO_2_ was fabricated, and SiO_2_ was deposited at the end on top of HfO_2_, as shown in the cross-sectional SEM image (Figure 5ii). The thicknesses of each layer were optimized and monitored via a quartz crystal microbalance. The present study demonstrated that one can design, tune, and fabricate AR coatings according to optical device requirements in the desired wavelength region just by optimizing thickness and utilizing material that has a suitable refractive index. In Table 3, E represents the thickness of the fabricated trilayer coating, and S shows the simulated thickness of an individual layer in a trilayer coating stack 

## 4. Conclusions

TFCalc helps and permits the operator to define an appropriate design using different inorganic materials applicable to various supporting substrates and illuminants. We used TFCalc thin film software to simulate AR coatings for optoelectronic devices. This software is a reliable simulation tool that can operate in varying environmental conditions. It helps in designing AR coatings according to one’s requirement. The optical behaviors of the monolayer, bilayer, and trilayer AR coatings on a silicon substrate are studied in detail. Experimental results showed that the single layer of hafnia is more suitable on a silicon substrate to enhance its AR efficiency compared to TiO_2_ or SiO_2_. In the case of bilayer coatings, stacking of SiO_2_-TiO_2_ is more applicable, compared to TiO_2_ and HfO_2_, because both of them are high-index materials. However, a trilayer coating comprising TiO_2_, HfO_2_, and SiO_2_ is the one which is beneficial in enhancing AR efficiency of silicon and reduces its reluctance from 36% to <3% in the visible region. This kind of coating can enhance solar cell productivity at omnidirectional angles because there is a gradual declination of the refractive index. Capturing of incident light is an essential requirement for high-performance, thin-film silicon solar cells. Photocurrent improvement in solar cells can be increased by fabricating these kinds of AR coatings, which are beneficial in transmitting maximum light in such a way that minimal light is reflected from the air–substrate interface.

## Figures and Tables

**Table 1:** Simulated constraints and experimentally measured efficiencies and thicknesses of monolayer inorganic oxide AR coatings on a silicon substrate.

	Material	Thickness	% Reflectance						
Wavelength	-	(nm)	300	400	500	550	600	700	800
	Silicon	-	50	39.34	35.32	35	33.77	33.42	33.16
Simulated	SiO_2_	85~90	33.92	17.79	10.13	10.41	11.71	14.51	19.60
Experimental		90	33.64	11.96	8.29	11.46	11.20	13.83	15.19
Simulated	HfO_2_	55~60	32.82	11.99	1.66	1.47	3.25	8.03	13.27
Experimental		57	33.39	8.70	2.02	1.55	2.85	5.91	9.80
Simulated	TiO_2_	40~50	31.38	10.61	3.22	4.81	7.72	12.98	18.02
Experimental		43	35.32	5.79	2.31	3.12	5.53	10.62	13,83

**Table 2:** Simulation parameters for modeling composite bilayer AR coatings.

	Material	Thickness		% Reflectance						
-	Wavelength (nm)	Layer 1	Layer 2	300	400	500	550	600	700	800
-	Silicon	-	-	50	39.34	35.32	35	33.77	33.42	33.16
Simulated	TiO_2_-HfO_2_	20~25	30~35	20.99	5.40	1.69	2.85	6.45	12.25	17.03
Experimental		20	30	19.66	3.53	2.28	3.28	6.73	11.48	14.40
Simulated	TiO_2_-SiO_2_	40~43	68~73	3.79	2.49	1.64	0.52	0.54	2.84	6.92
Experimental		40	60	8	1.21	0.33	1.41	2.1	0.08	4.48
Simulated	HfO_2_-SiO_2_	47~50	50~55	5.85	7.1	0.82	0.31	0.91	5.01	9.81
Experimental		45	50	3.7	5.1	2.20	0.62	1.66	4.32	8.82

**Table 3:** Measured and simulated AR efficiencies of trilayer AR coatings.

Material	Thickness E (nm) S (nm)		% Reflectance						
Wavelength (nm)			300	400	500	550	600	700	800
Silicon	No. of Layers		36.2	36.2	36.2	36.2	36.2	36.2	36.2
Layer 1-TiO_2_	35	36~40	3.2	2.17	1.90	0.33	0.33	2.17	6.45
Layer 2-HfO_2_	20	15~20	Experimental Measured Reflectance						
Layer 3-SiO_2_	70	58~60	2.97	0.33	0.03	0.44	0.80	0.22	1.16
